# E-Commerce and global dietary health: a longitudinal analysis of food availability and imports pathways across 64 countries (2008–2022)

**DOI:** 10.7189/jogh.15.04287

**Published:** 2025-08-29

**Authors:** Shiwen Quan, Huiyun Zhang

**Affiliations:** 1 Rural Development Institute, Chinese Academy of Social Sciences, Beijing, China; 2 Faculty of Applied Economics, University of Chinese Academy of Social Sciences, Beijing, China

## Abstract

**Background:**

Improving dietary health has become a key objective in public health policies worldwide. Concurrently, rapid technological advancements have profoundly altered food acquisition methods, with the widespread adoption of the internet shifting food commerce from offline to online platforms. This study aims to empirically examine the impact of e-commerce on residents' dietary health.

**Methods:**

Utilising unbalanced panel data from 64 countries between 2008 and 2022, this study employs a two-way fixed-effects model to empirically analyse the effects of e-commerce on dietary health. Mediation analysis is conducted to examine the underlying mechanisms, specifically food availability and food imports. Heterogeneity analysis is conducted to investigate the heterogeneity of these effects with different economic development levels.

**Results:**

E-commerce positively promotes dietary health among residents. Regarding specific dimensions of dietary health, the promotion effects of e-commerce on the Healthy Food Diet Index (HFD) and the Balanced Index of Food Consumption Structure (f_coi) are stronger than on the Balanced Index of Nutritional Structure (n_coi). Mechanism tests indicate that food availability acts as a partial mediating factor in the relationship between e-commerce and dietary health, while food imports mediate the relationship between e-commerce and two dietary health dimensions (HFD and f_coi) with indirect effect of 5.0% and 7.3% respectively. Heterogeneity analysis reveals that the positive impact of e-commerce on HFD and f_coi weakens progressively with rising levels of economic development.

**Conclusions:**

It is recommended that efforts should be made to strengthen supporting infrastructure such as cold chain logistics to effectively promote the positive impact of e-commerce on dietary health, especially in low-income countries. Concurrently, vigilant monitoring is required to mitigate the exacerbating effects of food imports on dietary health. The higher marginal benefit of e-commerce development on dietary health in low-income countries effectively narrows the dietary health gap with developed nations, thereby advancing global health equity.

A healthy diet contributes to the prevention of various forms of malnutrition and noncommunicable diseases (NCDs) such as diabetes, heart disease, stroke, and cancer. Unhealthy dietary practices and insufficient physical activity represent the greatest global health risks [1]. A refined study by the Food and Agriculture Organization of the United Nations (FAO) involving 156 countries confirms that hidden costs within global agrifood systems amount to approximately 12 trillion USD annually. Of this figure, around 70% (8.1 trillion USD) arise from unhealthy dietary patterns and are linked to alarming non-communicable diseases (NCDs) such as heart disease, stroke, and diabetes, far exceeding the costs related to environmental degradation and social inequalities [2]. Consequently, improving dietary health among residents has become a critical priority in the formulation of public health policies worldwide.

Meanwhile, rapid technological advancements have significantly transformed the ways people obtain food. The widespread adoption of the internet has gradually shifted food transactions from offline to online platforms. According to data from Statista, the global number of internet users has exceeded five billion, accompanied by a continuous increase in online shoppers. Global retail e-commerce sales are projected to surpass 4.3 trillion USD by 2025, with this figure expected to reach new heights in subsequent years [3]. E-commerce has become an indispensable component of global retail, profoundly reshaping food consumption choices. Specifically, e-commerce breaks down geographical barriers inherent in traditional food environments, enabling transactions between buyers and sellers separated by considerable distances. It substantially reduces transaction costs, including transportation and time expenses, and significantly expands consumers' food choices. From this perspective, e-commerce facilitates improvements in dietary diversity among consumers.

Therefore, an important question arises: does e-commerce effectively improve residents' dietary health? Currently, no empirical studies have been conducted using global cross-national samples to address this issue. Existing researches primarily examine how internet and mobile payment usage affect dietary health improvements residents of a single country [4–13]. Most of these studies provide evidence supporting the positive role of e-commerce in enhancing dietary health and have explored potential mechanisms, such as increased household income, improved information access skills, and reduced transaction costs for rural populations. However, two limitations are prominent in this body of literature. First, the findings apply exclusively to rural residents in China, whereas e-commerce inherently transcends geographic and national boundaries. Therefore, examining changes in dietary health across global populations in the context of e-commerce would yield a more comprehensive understanding of its overall impact, thereby assisting international organisations in formulating universally applicable macro policies. Second, existing studies predominantly employ micro-level dietary health metrics, such as the Healthy Eating Index (HEI), Dietary Diversity Score (DDS), and Dietary Rationality Score (DRS). These indices are counting-based micro-assessment tools, which not only fail to capture dietary health effectively at an aggregated macro level but also lack a strictly monotonic relationship with actual health outcomes. Consequently, they are insufficient for rigorously assessing dietary health status.

Based on the review of existing literature, this paper empirically investigates the impact of e-commerce on residents' dietary health using unbalanced panel data from 64 countries spanning the period 2008–2022. Furthermore, the study examines the mediating mechanisms of food availability and food imports, as well as the heterogeneity of these effects across regions with varying levels of economic development. Compared to prior research, this paper potentially offers marginal contributions in three key aspects. First, by conducting an empirical analysis using global cross-national data for the first time, the findings contribute to policy-making by international organisations aimed at improving dietary health through globally applicable e-commerce policies. Second, this paper employs more rigorous and scientifically valid measures of dietary health, thereby enhancing the credibility of the research outcomes. Third, the study explores, from a macro perspective, the mechanisms through which e-commerce influences dietary health – specifically, food availability and food imports – thereby expanding the scope of mechanism analyses in this research domain. Fourth, the mechanism section links the negative impact of e-commerce on food imports to the detrimental effect of food imports on dietary health. Our findings alert policymakers to the suppressive effect of e-commerce on food imports and the adverse health consequences of such imports, thereby informing preventive strategies. Simultaneously, they call for heightened academic attention to the underlying causes of these negative effects.

With the widespread adoption of information technologies and the rapid expansion of the global e-commerce market, e-commerce has gradually permeated various aspects of residents' lives, drawing particular attention in the field of food consumption [14,15]. The rise of e-commerce has substantially altered residents' food purchasing methods and dietary habits, consequently exerting significant impacts on their health [16,17]. On the one hand, online shopping reduces exposure to in-store stimuli as-sociated with appealing yet unhealthy foods, thereby decreasing spending on certain unhealthy, impulsive food choices. Grocery-based healthy diet programmes might leverage online ordering platforms to enhance their reach and effectiveness [18–20]. Harris-Lagoudakis [21] finds that online shopping results in healthier purchasing pat-terns compared to physical store shopping, with a 3.2 to 3.5 percentage point increase in budget or calorie share allocated to healthy product categories, higher nutritional density scores, and lower calorie density. On the other hand, e-commerce platforms can effectively disseminate nutritional information through digital technologies, enhancing residents' awareness of food quality and dietary health [22]. Zou and Liu [23] indicate that nutritional information significantly boosts online sales of healthy foods rather than unhealthy alternatives. Furthermore, from a supply perspective, e-commerce transcends traditional spatial and temporal constraints, allowing residents to conveniently purchase fresh and nutrient-rich foods anytime and anywhere, thus enhancing dietary diversity [5,17]. Accordingly, we propose:

Hypothesis 1. E-commerce positively contributes to residents' dietary health.

Dietary health is a combination of both the ‘quality’ and ‘quantity’ of food consumption, with ‘quantity’ serving as the foundation and prerequisite for ‘quality’. Generally speaking, ensuring sufficient ‘quantity’ is necessary for consumers to make rational choices to achieve an optimal ‘quality’ combination, thereby attaining the goal of healthy eating. Adequate food ‘quantity’ primarily relies on food supply, a concept deeply connected to food security. The definition of food security was first articulated: ‘Food security exists when all people, at all times, have physical and economic access to sufficient, safe and nutritious food to meet their dietary needs and food preferences for an active and healthy life’ [24]. This widely accepted definition highlights four critical dimensions:

1) food availability

2) food access

3) utilisation

4) stability.

Among these dimensions, the influence of e-commerce on food availability is the most prominent. Specifically, e-commerce platforms integrate supply and demand information, overcoming physical barriers to food transactions, and directly connecting producers and consumers [6,25,26]. Consumers can simultaneously access suppliers nationwide or even globally, significantly enriching both the quantity and variety of available foods [27,28], thus markedly enhancing food availability. Expanded food supply means consumers at the same economic level can obtain more di-verse food options. Increased dietary diversity typically leads to more balanced food consumption and nutritional structures, ultimately improving dietary health. Accordingly, we propose:

Hypothesis 2. E-commerce improves residents' dietary health by increasing food availability.

From a global perspective, the growth of e-commerce facilitates connections among national agricultural markets. Consumers in one country can now access food markets worldwide online, significantly broadening their range of food choices. Due to differences in resource endowments [29] and comparative advantages [30], countries specialise in exporting differentiated products, thus meeting consumers' diverse demands and consequently increasing a country's food imports. Studies have indicated that, concerning exports, e-commerce primarily enhances the quality of agricultural exports through optimising agricultural industry and supply chains, improving service levels, and aligning with consumer demand information [31]. Regarding imports, cross-border e-commerce has been shown to promote imports from developed countries, and primarily facilitate imports of clothing, food and daily necessities [32]. While improved ex-port quality primarily optimises food supply quality, increased food imports have a direct impact on the dietary health status of residents in importing countries. Furthermore, extensive research has confirmed the positive influence of trade on food security and dietary diversity [33–35]. However, some studies suggest that the relationship between imports and dietary health depends significantly on the types of food imported. Imports of fruits, vegetables, legumes, and nuts can mitigate dietary risks in importing countries, whereas imports of red meat can exacerbate dietary risks [36]. Some research even finds that increased trade leads to greater imports of processed foods and higher obesity rates [37,38].

While e-commerce reduces transaction costs and may initially boost food imports, the subsequent surge in import volumes can trigger regulatory arbitrage risks. This prompts governments to strengthen non-tariff barriers, ultimately suppressing import demand through compliance cost pass-through. Handley and Limao [39] demonstrate that trade policy uncertainty leads firms to reduce imports of high-risk categories, particularly price-elastic goods like food. Furthermore, e-commerce platforms streamline supply chains via direct procurement models but simultaneously activate spatial substitution (nearshoring) and production substitution (localisation), thereby bypassing traditional import channels [40]. Crucially, the transparency of e-commerce amplifies food safety incidents, eroding consumer trust. Li and Hitt [41] empirically validate that negative online reviews significantly impact sales, with each additional negative review reducing weekly sales. This mechanism aligns with Fiankor et al. [42] finding that health crises reduce food imports through importer attrition. Collectively, these findings suggest e-commerce may exert net negative effects on food imports.

Based on these considerations, the following hypotheses are proposed:

Hypothesis 3a. E-commerce improves residents' dietary health by increasing food imports.

Hypothesis 3b. E-commerce deteriorates residents' dietary health by increasing food imports.

Hypothesis 3c. E-commerce improves residents' dietary health by decreasing food imports.

Hypothesis 3d. E-commerce deteriorates residents' dietary health by decreasing food imports.

## METHODS

### Data analysis

The core independent variable in this study is the e-commerce penetration rate, with e-commerce retail sales serving as a robustness check indicator; the data for these variables are sourced from the eMarketer database. The dependent variable is the dietary health status of residents, composed of three indicators: the Healthy Food Diversity Index (HFD), the Balanced Index of Nutritional Structure (n_coi), and the Balanced Index of Food Consumption Structure (f_coi). These indicators are constructed from actual per capita food consumption data and recommended food intake levels from dietary guidelines, both sourced from the Food and Agriculture Organization of the United Nations (FAO). Matching samples from independent and dependent variables resulted in an unbalanced panel data set covering 64 countries for the period 2008–2022.

Moreover, considering the potential impacts of per capita GDP, road density, female population ratio, education level, urbanisation level, income inequality, health care infrastructure, industry, population density and agriculture on dietary health, this study incorporates these four factors as control variables, with data obtained from the World Bank. We further incorporate controls for Consumer Prices Indices and the Agricultural Trade Openness Index (FAO data). To address potential endogeneity concerns arising from omitted variables and reverse causality, two instrumental variables are introduced [6,43,44]: ease of starting a business (startbusiness) and Plain area(landrate)with data sourced from the World Bank. As the Plain area instrumental variables remain constant over time, we additionally construct an interaction term between the variable and Internet penetration rate with a one-period lag. This interaction term serves as an instrumental variable for two-stage least squares estimation.

The mediating variables explored in this research include the food availability and food imports. The food availability data are sourced from the Global Food Security Index (GFSI), and the food imports data are obtained from FAO. Descriptive statistical results for all the variables described above are presented (**Table 1**).

**Table 1:** Variables and descriptive statistics

Variable	Variable definition	N	Mean	SD	Min	Max
HFD	The Healthy Food Diversity Index	587	0.89	0.08	0.66	1.00
n_coi	The Balanced Index of Nutritional Structure; similarity of nutritional structure with dietary guidelines	587	0.85	0.04	0.75	0.98
f_coi	The Balanced Index of Food Consumption Structure; similarity of food consumption structure with dietary guidelines	587	0.78	0.08	0.57	0.97
EcomP	Retail Ecommerce Sales Penetration: % of total retail sales	587	0.06	0.06	0.00	0.46
ln1RES*	Retail Ecommerce Sales (billion USD)	587	3.90	2.03	0.21	10.15
lnincome†	GDP per capita (constant 2015 USD)	587	9.69	0.98	7.18	11.41
Railper	Railroad density	587	0.03	0.04	0.00	0.22
female	Population, female (% of total population)	587	50.19	3.59	24.62	54.89
School	School enrolment, tertiary (% gross)	587	66.28	24.69	14.82	166.67
urban	Urban population (% of total population)	523	72.91	15.79	29.51	104.90
Gini	Gini index	397	36.56	6.86	23.20	55.10
foodcpi	Consumer prices, food indices (2015 = 100)	587	111.06	52.06	56.25	1076.20
Hospital	Hospital beds (per 1000 people)	380	3.72	2.81	0.43	13.71
Industry	Industry (including construction), value added (% of GDP)	568	26.61	8.62	5.99	65.35
Population	Population density (people per km^2^ of land area)	587	143.71	149.87	2.87	652.07
agriopen	Agricultural trade openness index	538	0.07	0.04	0.01	0.22
Agriculture	Agriculture, forestry, and fishing, value added (% of GDP)	568	4.56	4.12	0.05	20.41
startbusiness	Starting a business – score	370	86.27	9.26	58.68	99.98
landrate	Land area where elevation is below five metres (% of total land area)	572	2.99	3.66	0.00	15.94
InterP	Individuals using the Internet (% of population)	570	74.70	19.08	10.07	100.00
Food availability	Availability index of GFSI	463	63.01	8.00	40.00	81.70
Food imports	Real Per Capita Food Imports (excluding fish) in constant 2015 prices	587	0.67	4.13	0.00	31.18

GDP – gross domestic product, SD – standard deviation

*The variable has been log-transformed after adding 1. This adjustment was necessary because the original variable exhibits pronounced long-tail characteristics, deviates from normality, and contains values less than 1.

†The variable has undergone a direct logarithmic transformation.

### Study design

This study employs a two-way fixed effects model to estimate the impact of e-commerce penetration rates on residents' dietary health status. The econometric model specification is presented in Equation 1:



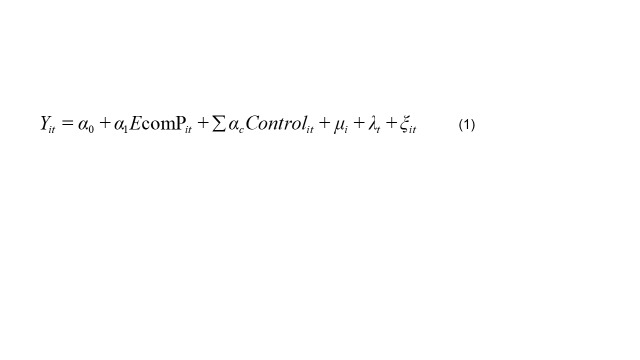

(1)


The subscripts *i*, *t* represent the country and year, respectively. The dependent variable *Y_it_* comprises the Healthy Food Diversity Index (*HGD_it_*), the Balanced Index of Food Consumption Structure (*f_coi_it_*), which compares actual per capita food consumption to dietary guidelines, and the Balanced Index of Nutritional Structure (*n_coi_it_*), similarly comparing actual per capita nutrient intake with dietary guidelines. *Control_it_* denotes control variables, including ln*income_it_*, *Railper_it_, female_it_, School_it_, urban_it_, Gini_it_, foodcpi_it_, Hospital_it_, Industry_it_, Population_it_, agriopen_it_, Agriculture_it_*. *μ_i_* represents country fixed effects, *λ_t_* denotes year fixed effects, *ξ_it_* is the random error term.



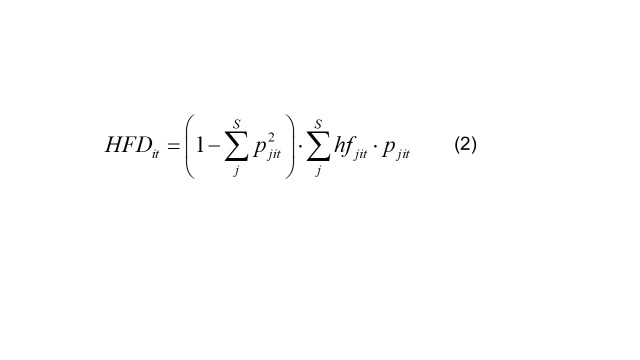

(2)




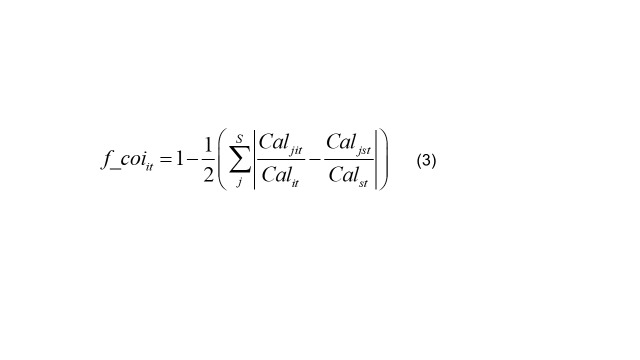

(3)




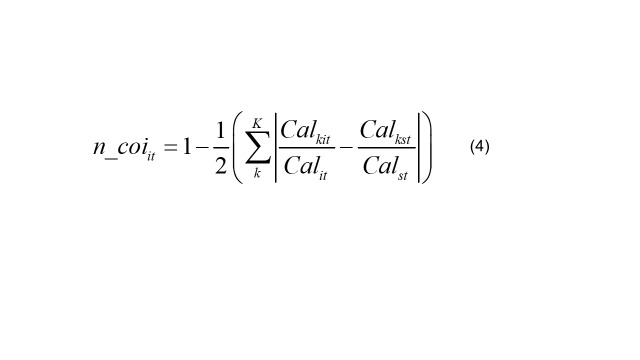

(4)


The three indicators of the dependent variable are represented Equations 2, 3, and 4. The subscript *j* denotes the food category, *j* = 1,2,3,‧‧‧,*S*, while the subscript *k* indicates the nutrient category, *k* = 1,2,3,‧‧‧*K*. *HFDit* is an index proposed by Drescher et al. [45], based on the Simpson Index, where *hf_jit_* represents the health factor of the *j* food category for country *i* in year *t*, calculated according to the dietary guidelines' recommended shares of various food categories as presented in Equation 2. The HFD-Index, which is bounded between 0 and 1–1/n, has the following desired properties:

1) If the distribution between hf groups of the pyramid does not change, it increases with the growing number of food items; it increases the more equally the food items are distributed within the hf-groups; and

2) If distribution between hf groups of the pyramid does change in favour of healthy (unhealthy) food groups, it increases (decreases).

It calculates the structural similarity between the caloric consumption of various food categories for country *i* in year *t* and the recommended caloric consumption according to the national dietary guidelines *s*, using Manhattan distance, followed by Schmidhuber and Traill [46] as presented in Equation 3. Given that *cal* = *Ʃ_j_cal_j_*, the range of *f_co_it_* is (0,1). *f_co_it_* measures the proportion of overlapping food sources in the dietary structures of country *i* and the national dietary guidelines *s*. A higher value indicates greater similarity, implying a healthier dietary structure in country *i*. Specifically, *f_coi_it_ =* 1 if country *i’s* consumption structure perfectly matches the dietary guidelines, and *f_coi_it_* = 0 if there is no overlap. It calculates the structural similarity between caloric intake from various nutrient categories and the caloric intake recommended by the national dietary guidelines for country *i* in year *t*, presented in Equation 4.

To examine the mediating roles of food availability and food imports in the causal relationship between e-commerce and residents' dietary health, this study follows the mediation effect model framework proposed by Mackinnon and Dwyer [47], as specified below:



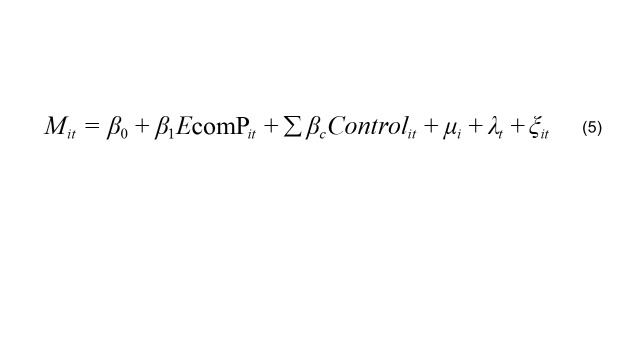

(5)




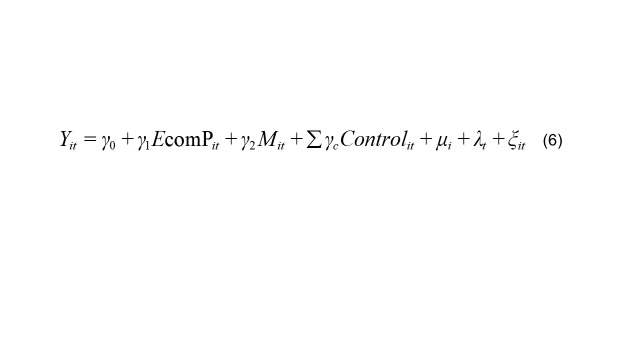

(6)


They together form the complete mediation effect testing model, as presented in Equations 1, 5, and 6. *M_it_* denotes the mediating variables, including food availability (*food availability_it_*) and the proportion of food imports (*food import_it_*). If *β_1_* is significant, *γ_2_* is significant, and *γ_1_* is not significant, this indicates the presence of a full mediation effect. If β_1_, γ_2_, and γ_1_ are all significant, it suggests the presence of a partial mediation effect.

## RESULTS

### Benchmark regression results

This paper first investigates the impact of e-commerce on dietary health with the benchmark regression results presented (**Table 2**). To test the robustness of the benchmark regression results, this paper replaces the core independent variable – e-commerce penetration rate with e-commerce retail sales (ln1RES). Given the long-tail distribution of e-commerce retail sales, which deviates from normality, the variable is log-transformed. Additionally, since the value of this variable is less than 1 in some cases, 1 is added to all observations prior to taking the logarithm. The coefficient on e-commerce penetration rate and e-commerce retail sales remains statistically significant at the 1% level (**Table 2**). This indicates that the results are highly robust, thereby lending strong support to Hypothesis 1.

**Table 2:** Benchmark regression results*

Variables	HFD	f_coi	n_coi	HFD	f_coi	n_coi
	(1)	(2)	(3)	(4)	(5)	(6)
EcomP	0.571† (0.380, 0.763)	0.385† (0.210, 0.560)	0.307† (0.198, 0.416)			
ln1RES				0.046† (0.029, 0.064)	0.035† (0.021, 0.050)	0.017† (0.007, 0.027)
lnincome	−0.037 (0.123, 0.050)	−0.044 (0.131, 0.042)	0.050‡ (0.006, 0.106)	−0.043 (−0.126, 0.039)	−0.052 (−0.141, 0.038)	0.052‡ (−0.006, 0.110)
Railper	−0.486 (3.105, 2.133)	−0.460 (−2.707, 1.787)	0.252 (−1.293, 1.798)	−1.571 (−4.007, 0.866)	−1.268 (−3.436, 0.900)	−0.185 (−1.614, 1.243)
Female	0.049† (0.022, 0.076)	0.030§ (0.003, 0.058)	0.037† (0.018, 0.055)	0.053† (0.026, 0.081)	0.034§ (0.006, 0.062)	0.038† (0.019, 0.057)
School	−0.001† (−0.002, −0.001)	−0.001† (−0.002, −0.001)	−0.000§ (−0.001, −0.000)	−0.001§ (−0.002, −0.000)	−0.001† (−0.002, −0.000)	−0.000 (−0.001, 0.000)
Urban	0.005‡ (−0.001, 0.011)	0.004 (−0.001, 0.009)	0.004§ (0.001, 0.007)	0.002 (−0.004, 0.008)	0.001 (−0.004, 0.007)	0.003 (−0.001, 0.006)
Gini	0.000 (−0.004, 0.004)	−0.001 (−0.005, 0.003)	0.001 (−0.002, 0.003)	0.001 (−0.003, 0.004)	−0.000 (−0.004, 0.004)	0.001 (−0.002, 0.003)
Foodcpi	−0.000 (−0.000, 0.000)	0.000‡ (−0.000, 0.000)	0.000 (−0.000, 0.000)	−0.000 (−0.000, 0.000)	0.000 (−0.000, 0.000)	0.000 (−0.000, 0.000)
Hospital	−0.014 (−0.033, 0.006)	−0.012 (−0.034, 0.010)	−0.008 (−0.022, 0.006)	−0.031† (−0.050, −0.011)	−0.025§ (−0.048, −0.002)	−0.015§ (−0.029, −0.000)
Industry	−0.000 (−0.004, 0.003)	0.000 (−0.003, 0.003)	0.00 (−0.001, 0.002)	0.000 (−0.003, 0.004)	0.000 (−0.003, 0.003)	0.001 (−0.001, 0.003)
Population	0.001§ (0.000, 0.002)	0.001† (0.000, 0.002)	0.001† (0.001, 0.002)	−0.001 (−0.002, 0.000)	−0.000 (−0.001, 0.001)	0.000 (−0.000, 0.001)
agriopen	−0.065 (−0.516, 0.386)	−0.078 (−0.535, 0.379)	−0.048 (−0.375, 0.279)	−0.138 (−0.583, 0.306)	−0.115 (−0.549, 0.318)	−0.110 (−0.456, 0.236)
Agriculture	0.005 (−0.002, 0.012)	0.002 (−0.005, 0.008)	0.004‡ (−0.000, 0.009)	0.005 (−0.001, 0.012)	0.002 (−0.005, 0.008)	0.005§ (0.000, 0.010)
_cons	−1.668 (−3.770, 0.435)	−0.647 (−2.670, 1.376)	−1.912† (−3.157, −0.667)	−1.450 (−3.487, 0.587)	−0.461 (−2.492, 1.570)	−1.870† (−3.162, −0.579)
Country FE	Yes	Yes	Yes	Yes	Yes	Yes
Year FE	Yes	Yes	Yes	Yes	Yes	Yes
Observations	283	283	283	283	283	283
Adj R2	0.918	0.916	0.892	0.921	0.919	0.887

*95% confidence interval (CI) is shown in parenthesis.

†Denotes that the coefficient is significant at 1% (*P* < 0.01).

‡Denotes that the coefficient is significant at 10% (*P* < 0.1).

§Denotes that the coefficient is significant at 5% (*P* < 0.05).

The impact of e-commerce penetration rate on the three indicators of dietary health exhibits notable differences. According to the estimation results from the two-way fixed effects model, the marginal effects of e-commerce penetration rate are statistically significant and positive across all three measures of dietary quality. For the Healthy Food Diversity Index (HFD), the marginal effect is 0.571 (95% confidence interval (CI) = 0.380, 0.763). For the Balanced Index of Food Consumption Structure (f_coi), the marginal effect is 0.385 (95%CI = 0.210, 0.560). For the Balanced Index of Nutritional Structure (n_coi), the marginal effect is 0.307 (95%CI = 0.198, 0.416). These results indicate that the effect of e-commerce on improving dietary diversity is almost than twice as large as its effect on improving nutritional structure. Besides, the effect of e-commerce on improving food consumption structure is larger than nutritional structure. A plausible explanation is that e-commerce primarily expands the availability and variety of food, thereby enhancing dietary diversity among residents. This, in turn, contributes to a more balanced food consumption structure. However, a balanced variety of food types does not necessarily translate into an equally balanced nutritional structure, as different food categories may contain similar nutritional elements despite their apparent variety.

### Results of the IV regressions

Given the potential endogeneity issues in the baseline regression, such as omitted variable bias and reverse causality, this study addresses the problem by employing an instrumental variable (IV) approach. Following the methodologies of Djankov [43], Shen et al. [6], and Tang et al. [44], we select two instrumental variables: the annual ease of starting a business and the percentage of land area below five meters in elevation for each country. Given the time-invariant nature of the latter variable across years, we construct our second instrument as its interaction term with the one-year lagged internet penetration rate. Theoretically, both instruments satisfy the requirements of relevance and exclusion restriction. The ease of starting a business serves as a core indicator of a nation's business environment, regulatory efficiency, and market accessibility. Lower entry barriers directly foster the proliferation and competitiveness of the e-commerce ecosystem, while higher regulatory efficiency reduces compliance costs for business operations, including online activities, thereby promoting e-commerce development and ensuring relevance. Crucially, it is posited not to exert a direct influence on residents' dietary health; its impact is channelled primarily through stimulating the e-commerce sector itself, rather than through broader economic development pathways, satisfying the exclusion restriction. The inherent advantages of low-lying areas (*e.g*. market concentration, logistics efficiency) amplify the promotional effect of internet penetration on e-commerce. Simultaneously, internet penetration enhances the potential for e-commerce growth in these low-elevation regions. This instrument is theoretically relevant to e-commerce activity. However, it is unlikely to directly affect the dietary health of residents in the focal country, thus satisfying both the relevance and exclusion assumptions. The instrumental variable analysis is conducted using the two-stage least squares (2SLS) method (**Table 3**).

**Table 3:** 2SLS estimates using instrumental variables*

Variables	First stage	Second stage		
	(1)	(2)	(3)	(4)
	EcomP	HFD	f_coi	n_coi
EcomP		0.554† (0.091, 1.017)	0.396‡ (−0.059, 0.851)	0.434† (0.083, 0.785)
L2.startbusiness	0.002§ (0.001, 0.003)			
L.InterP × landrate	0.000† (0.000, 0.001)			
lnincome	0.009 (−0.080, 0.098)	−0.067 (−0.216, 0.083)	−0.062 (−0.198, 0.075)	0.024 (−0.075, 0.122)
Railper	−1.627 (−3.785, 0.532)	−1.349 (−4.864, 2.166)	−1.553 (−4.704, 1.598)	0.072 (−2.318, 2.462)
Female	−0.016 (−0.045, 0.013)	0.022 (−0.023, 0.071)	0.019 (−0.028, 0.066)	0.035† (0.006, 0.065)
School	0.000 (−0.000, 0.001)	−0.001† (−0.002, −0.000)	−0.001§ (−0.002, −0.001)	−0.000‡ (−0.001, 0.000)
Urban	−0.005‡ (−0.011, 0.000)	0.006 (−0.005, 0.016)	0.004 (−0.005, 0.013)	0.003 (−0.002, 0.007)
Gini	−0.001 (−0.004, 0.001)	−0.003 (−0.009, 0.003)	−0.002 (−0.008, 0.004)	0.000 (−0.003, 0.004)
Foodcpi	0.000 (−0.000, 0.000)	−0.000 (−0.000, 0.000)	0.000 (−0.000, 0.000)	−0.000 (−0.001, 0.000)
Hospital	−0.034† (−0.062, −0.007)	0.000 (−0.039, 0.040)	0.011 (−0.030, 0.053)	0.006 (−0.021, 0.034)
Industry	0.001 (−0.001, 0.003)	−0.002 (−0.007, 0.004)	−0.003 (−0.007, 0.002)	−0.001 (−0.004, 0.002)
Population	−0.001§ (−0.002, −0.000)	0.000 (−0.001, 0.002)	0.000 (−0.001, 0.002)	0.001‡ (−0.000, 0.002)
agriopen	−0.412† (−0.749, −0.075)	0.262 (−0.393, 0.917)	0.203(−0.417, 0.824)	0.053 (−0.383, 0.489)
Agriculture	0.003 (−0.004, 0.010)	0.005 (−0.005, 0.015)	0.002 (−0.007, 0.011)	0.003 (−0.003, 0.010)
Country FE	Yes	Yes	Yes	Yes
Year FE	Yes	Yes	Yes	Yes
Kleibergen-Paap rk LM statistic	6.425 (0.040)			
Cragg-Donald Wald F statistic	30.357			
Hansen J statistic		1.327 (0.249)	2.179 (0.140)	0.000 (0.996)
Endogeneity test		0.049 (0.825)	0.003 (0.955)	2.492 (0.114)
Observations	190	190	190	190
R2		0.238	0.203	0.258

*95% confidence interval (CI) are shown in parenthesis.

†Denotes that the coefficient is significant at 5% (*P* < 0.05).

‡Denotes that the coefficient is significant at 10% (*P* < 0.1).

§Denotes that the coefficient is significant at 1% (*P* < 0.01).

The diagnostic results indicate that the Kleibergen-Paap rk LM statistic significantly rejects the null hypothesis of underidentification, suggesting that the instruments are correlated with the endogenous explanatory variable. The Cragg-Donald Wald F statistic is well above 10, indicating that the model does not suffer from weak instrument problems. Moreover, the Hansen J statistic fails to reject the joint null hypothesis that the instruments are valid, confirming their exogeneity. The regression results for the effects of e-commerce penetration rate (EcomP) are as follows: for the HFD, the coefficient is 0.554 (95% CI = 0.091, 1.017). For the Balanced Index of Food Consumption Structure (f_coi), the coefficient is 0.396 (95% CI = −0.059, 0.851). For the Balanced Index of Nutritional Structure (n_coi), the coefficient is 0.434 (95% CI = 0.083, 0.785). Compared to the baseline results (**Table 2**), these coefficients have changed by -2.98, 2.86, and 41.37%, respectively. The empirical findings are consistent with the baseline results. It is noteworthy that the Endogeneity test indicates the baseline model does not suffer from endogeneity issues. These results confirm that the main conclusions support for Hypothesis 1.

## DISCUSSION

### Mechanism I: food availability

To further examine the mediating role of food availability in the process through which e-commerce promotes residents’ dietary health, this study conducts empirical tests (Table S1 in the **Online Supplementary Document**). The baseline regression results indicate that the effects of EcomP on HFD, f_coi, and n_coi are all significantly positive at the 1% statistical level. The second step of mediation analysis reports the promoting effect of EcomP on food availability is 25.629 (95% CI = −0.965, 52.222) and statistically significant. The results display the third step of mediation analysis: while the regression coefficients of food availability on HFD, f_coi, and n_coi are positive, they lack statistical significance. This insignificance may arise from high correlation between EcomP and food availability, which inflates the standard errors of food availability’s coefficients. Furthermore, controlling for post-treatment variables constitutes ‘bad control’, and including mediating variables represents a typical case of Bad Control [48]. Additionally, the impact of food availability on dietary health is robustly supported by theoretical and empirical studies [49–51]. The regression coefficients of EcomP are as follows: 0.488 (95%CI = 0.291, 0.686) on HFD, 0.359 (95% CI = 0.162, 0.556) on f_coi, and 0.309 (95% CI = 0.190, 0.427) on n_coi. These coefficients are lower than those yet still significant at the 1% level – demonstrating that food availability partially mediates the positive effects of EcomP on HFD, f_coi, and n_coi. This finding aligns with conclusions from Cummins et al. [52], Rogus et al. [53], and Mah et al. [54]. These results confirm that EcomP promotes Availability, thereby improving dietary health, thus validating Hypothesis 2.

### Mechanism II: food imports

It examines the mediating role of food imports in the relationship between e-commerce penetration rate (EcomP) and residents’ dietary health (Table S2 in the **Online Supplementary Document**). The second step of the mediation analysis shows that the coefficient of EcomP on food imports is −0.003 (95% CI = −0.007, 0.001), which is statistically significant at the 5% level. The third step of the mediation analysis reports the following estimated effects of EcomP: 0.541 (95% CI = 0.347, 0.734) on HFD, 0.355 (95% CI = 0.178, 0.532) on f_coi, and 0.299 (95% CI = 0.190, 0.408) on n_coi. Compared with the corresponding coefficients in the benchmark regression results (**Table 2**), these values have decreased but remain statistically significant at the 1% level. This indicates that food imports partially mediate the positive impact of EcomP on HFD, f_coi, and n_coi, as EcomP reduces food imports thereby diminishing their negative effects on dietary health. This finding is in line with the findings of Cassels [36] and Law [37]. Further calculation reveals that the indirect effects of EcomP through food imports on HFD and f_coi are 5.0% and 7.3%, respectively. These findings suggest that EcomP reduces food imports, which in turn improves HFD and f_coi. Therefore, Hypothesis 3c is supported.

### Heterogeneous effects with different levels of economic development

To examine whether the impact of e-commerce penetration rate (EcomP) on residents’ dietary health varies across regions with different economic development levels, this study incorporates an interaction term between lnincome and EcomP into Equation 1 (Table S3 in the **Online Supplementary Document**). The results indicate that economic development level exerts a negative moderating effect on the relationship between EcomP and dietary health. In models with HFD and f_coi as dependent variables, the coefficients of the lnincome × EcomP interaction term are as follows: −0.211 (95% CI = −0.379, −0.043), statistically significant at the 5% level for HFD; −0.220 (95% CI = −0.378, −0.063), statistically significant at the 1% level for f_coi. For the n_coi model, the interaction term coefficient is -0.065 but lacks statistical significance. These findings demonstrate a gradient attenuation pattern in EcomP’s effects on HFD and f_coi as economic development advances. A plausible explanation is that high-income regions have already optimised food consumption through income-driven factors and developed food environments, resulting in relatively mature dietary patterns where the marginal benefits of EcomP in promoting dietary health are considerably reduced [55]. In this context, the low sensitivity of food consumption to e-commerce in high-income countries parallels its low responsiveness to income changes, consistent with Seale et al. [56] who found differential responses to income and food price changes across income groups, with greater sensitivity in low-income nations.

## CONCLUSIONS

Based on an unbalanced panel data set covering 64 countries from 2008 to 2022, this study empirically investigates the impact of e-commerce on residents’ dietary health. It further examines the underlying mechanisms through which food availability and food imports operate, as well as the heterogeneity of effects across regions with different levels of economic development. The main findings are as follows:

First, e-commerce positively contributes to improving dietary health. Among the sub-dimensions of dietary health, the effects of e-commerce penetration rate (EcomP) on the Healthy Food Diversity Index (HFD) and the Balanced Index of Food Consumption Structure (f_coi) are stronger than its effect on the Balanced Index of Nutritional Structure (n_coi). Second, food availability serves as a partial mediating channel in the relationship between e-commerce and dietary health, while food imports mediates the relationship between e-commerce and two dietary health dimensions (HFD and f_coi) with indirect effect of 5.0 and 7.3% respectively. Third, EcomP suppresses food imports via localisation substitution effects. This attenuation mechanism significantly mitigates the adverse impacts of food imports on HFD and f_coi. Fourth, the marginal effects of EcomP on HFD and f_coi exhibit diminishing returns with economic development advancement, conforming to a gradient attenuation pattern.

The above findings offer significant implications for countries aiming to improve residents’ dietary health through e-commerce policies. First, governments should optimise the domestic e-commerce business environment, encourage enterprises to engage in online sales, and enhance residents’ digital skills and e-commerce purchasing habits, thereby enabling e-commerce to effectively promote healthier diet. Second, efforts should be made to strengthen supporting infrastructure such as cold chain logistics to ensure the effective functioning of the food availability mechanism. Additionally, vigilant monitoring is required to mitigate the exacerbating effects of food imports on dietary health. Third, differentiated management strategies should be adopted based on the level of economic development. Low-income countries should prioritise e-commerce development during this strategic window of opportunity, given the diminishing marginal returns of e-commerce on dietary health at higher development levels. The higher marginal benefit of e-commerce development on dietary health in low-income countries effectively narrows the dietary health gap with developed nations, thereby advancing global health equity.

We propose the establishment of a refined food health classification and risk rating system integrated with cross-border e-commerce platforms. This system should mandate platforms to implement compulsory electronic health labeling (*e.g*. Nutri-Score, warning labels) for imported food products. Health risk ratings should be dynamically assigned based on key nutritional indicators such as sugar content, saturated fat, and sodium levels. For high-risk (unhealthy) food categories imported via e-commerce channels, the implementation of an additional ‘Public Health Levy’ or ‘Health Impact Surcharge’ is recommended, alongside the imposition of platform-level purchase quantity limits (per transaction and/or annually). Concurrently, a streamlined ‘Green Channel’ should be created to facilitate the import of certified low-risk healthy foods (*e.g*. specific fresh fruits, whole grains, low-fat dairy products) through cross-border e-commerce. Recommended measures include:

1) simplification of customs declaration procedures,

2) reduction in inspection and quarantine sampling rates,

3) expedited customs clearance,

4) exploration of tariff reductions or exemptions for specific healthy food categories. Furthermore, platforms should be encouraged to establish dedicated ‘Healthy Food Cross-Border Zones’ and provide enhanced transparency regarding product origin, certifications, and logistics information.

## Additional material


Online Supplementary Document
