# Supplementary material for: E-Commerce and global dietary health: a longitudinal analysis of food availability and imports pathways across 64 countries (2008–2022)
Source: J Glob Health. 2025 Aug 29;15:04287. doi: 10.7189/jogh.15.04287 (PMC12396156; doi:10.7189/jogh.15.04287)
Supplement: Online Supplementary Document [file jogh-15-04287-s001.pdf]

Supplement to: Quan S, Zhang H. E-Commerce and global dietary health: a longitudinal analysis of food availability and imports pathways across 64 countries (2008–2022). J Glob Health. 2025;15:04287.

Table S1. Results of the analysis of the mechanism of action of food availability

	HFD	f_coi	n_coi	Food availability	HFD	f_coi	n_coi
	(1)	(2)	(3)	(4)	(5)	(6)	(7)
EcomP	0.504*** (0.308, 0.700)	0.368*** (0.174, 0.562)	0.316*** (0.197, 0.435)	25.629* (-0.965, 52.222)	0.488*** (0.291, 0.686)	0.359*** (0.162, 0.556)	0.309*** (0.190, 0.427)
Food availability					0.001 (-0.000, 0.002)	0.000 (-0.001, 0.001)	0.000 (-0.000, 0.001)
lnincome	0.009 (-0.087, 0.105)	-0.001 (-0.109, 0.107)	0.075** (0.009, 0.142)	-18.062** (-35.594, -0.530)	0.020 (-0.078, 0.118)	0.005 (-0.104, 0.115)	0.080** (0.013, 0.148)
Railper	-0.635 (-3.089, 1.818)	-0.682 (-2.865, 1.501)	0.141 (-1.364, 1.647)	-143.643 (-838.844, 551.558)	-0.550 (-2.951, 1.852)	-0.631 (-2.814, 1.552)	0.181 (-1.264, 1.626)
female	0.013 (-0.028, 0.053)	0.003 (-0.043, 0.049)	0.019 (-0.009, 0.046)	-0.526 (-6.442, 5.390)	0.013 (-0.028, 0.054)	0.003 (-0.043, 0.049)	0.019 (-0.009, 0.047)
School	-0.001*** (-0.002, -0.001)	-0.001*** (-0.002, -0.001)	-0.000 (-0.001, 0.000)	-0.267*** (-0.392, -0.143)	-0.001** (-0.002, -0.000)	-0.001*** (-0.002, -0.001)	-0.000 (-0.001, 0.000)
urban	0.005* (-0.001, 0.011)	0.004 (-0.002, 0.009)	0.004** (0.001, 0.007)	1.408*** (0.483, 2.332)	0.004 (-0.002, 0.011)	0.003 (-0.002, 0.009)	0.004** (0.000, 0.007)
Gini	-0.001 (-0.005, 0.003)	-0.002 (-0.006, 0.003)	-0.000 (-0.003, 0.002)	-0.567* (-1.214, 0.080)	-0.000 (-0.004, 0.004)	-0.001 (-0.006, 0.003)	-0.000 (-0.003, 0.002)
foodcpi	-0.000 (-0.000, 0.000)	0.000 (-0.000, 0.000)	-0.000 (-0.000, 0.000)	0.017* (-0.001, 0.035)	-0.000 (-0.000, 0.000)	0.000 (-0.000, 0.000)	-0.000 (-0.000, 0.000)
Hospital	-0.010 (-0.030, 0.010)	-0.014 (-0.038, 0.010)	-0.014** (-0.028, -0.000)	0.186 (-3.652, 4.023)	-0.010 (-0.030, 0.010)	-0.014 (-0.038, 0.010)	-0.014** (-0.028, -0.001)
Industry	-0.001 (-0.005, 0.003)	-0.001 (-0.005, 0.002)	-0.000 (-0.002, 0.002)	-0.233 (-0.910, 0.444)	-0.001 (-0.005, 0.003)	-0.001 (-0.005, 0.002)	-0.000 (-0.002, 0.002)
Population	0.000 (-0.001, 0.001)	0.001 (-0.000, 0.002)	0.001*** (0.000, 0.002)	0.029 (-0.116, 0.174)	0.000 (-0.001, 0.001)	0.001 (-0.000, 0.002)	0.001** (0.000, 0.002)
agriopen	0.366 (-0.157, 0.889)	0.247 (-0.322, 0.816)	0.129 (-0.239, 0.498)	51.779 (-55.362, 158.921)	0.335 (-0.189, 0.859)	0.229 (-0.340, 0.797)	0.115 (-0.254, 0.484)
Agriculture	0.005 (-0.002, 0.012)	0.003 (-0.004, 0.010)	0.004* (-0.000, 0.009)	-1.580*** (-2.768, -0.392)	0.006* (-0.001, 0.013)	0.004 (-0.003, 0.011)	0.005** (0.000, 0.010)

_cons	-0.154 (-2.933, 2.626)	0.466 (-2.510, 3.442)	-1.191 (-2.964, 0.581)	203.919 (-156.203, 564.042)	-0.275 (-3.020, 2.469)	0.394 (-2.555, 3.343)	-1.248 (-3.036, 0.540)
Country FE	Yes	Yes	Yes	Yes	Yes	Yes	Yes
Year FE	Yes	Yes	Yes	Yes	Yes	Yes	Yes
Observations	238	238	238	238	238	238	238
Adj R2	0.912	0.892	0.885	0.833	0.912	0.892	0.885

95% confidence interval (CI) are shown in parenthesis.
*denotes that the coefficient is significant at 10%(p<0.1); ** at 5%(p<0.05) and *** at 1%(p<0.01).

Table S2. Results of the analysis of the mechanism of action of food imports

	**Food imports**	**HFD**	**f_coi**	**n_coi**
	(1)	(2)	(3)	(4)
EcomP	-0.003* (-0.007,0.001)	0.541*** (0.347,0.734)	0.355*** (0.178,0.532)	0.299*** (0.190,0.408)
Food imports		-9.540*** (-16.583,-2.496)	-9.281*** (-15.066,-3.495)	-2.606 (-6.386,1.174)
lnincome	0.005*** (0.003,0.006)	0.006 (-0.083,0.095)	-0.003 (-0.091,0.086)	0.062** (0.004,0.119)
Railper	-0.035 (-0.093,0.023)	-0.823 (-3.268,1.623)	-0.788 (-2.887,1.312)	0.160 (-1.394,1.715)
female	0.000 (-0.000,0.001)	0.052*** (0.026,0.079)	0.033** (0.006,0.061)	0.037*** (0.019,0.056)
School	0.000* (-0.000,0.000)	-0.001*** (-0.002,-0.000)	-0.001*** (-0.002,-0.001)	-0.000* (-0.001,0.000)
urban	-0.000 (-0.000,0.000)	0.005* (-0.001,0.011)	0.004 (-0.001,0.009)	0.004** (0.001,0.007)
Gini	-0.000 (-0.000,0.000)	0.000 (-0.004,0.004)	-0.001 (-0.005,0.003)	0.000 (-0.002,0.003)
foodcpi	-0.000*** (-0.000,-0.000)	-0.000 (-0.000,0.000)	0.000 (-0.000,0.000)	0.000 (-0.000,0.000)
Hospital	-0.000** (-0.001,-0.000)	-0.018* (-0.036,0.001)	-0.016 (-0.038,0.005)	-0.009 (-0.023,0.005)
Industry	-0.000** (-0.000,-0.000)	-0.001 (-0.004,0.003)	-0.001 (-0.004,0.002)	0.000 (-0.002,0.002)
Population	-0.000*** (-0.000,-0.000)	0.001* (-0.000,0.002)	0.001** (0.000,0.002)	0.001*** (0.000,0.002)
agriopen	0.016** (0.003,0.029)	0.089 (-0.352,0.530)	0.072 (-0.376,0.520)	-0.006 (-0.327,0.316)
Agriculture	-0.000 (-0.000,0.000)	0.005 (-0.002,0.012)	0.002 (-0.005,0.008)	0.004* (-0.000,0.009)
_cons	-0.045** (-0.083,-0.007)	-2.097** (-4.128,-0.067)	-1.065 (-3.034,0.903)	-2.030*** (-3.289,-0.770)
Country FE	Yes	Yes	Yes	Yes
Year FE	Yes	Yes	Yes	Yes

Observations	283	283	283	283
Adj R2	0.994	0.920	0.918	0.892

95% confidence interval (CI) are shown in parenthesis.
*denotes that the coefficient is significant at 10%(*p*<0.1); ** at 5%(*p*<0.05) and *** at 1%(*p*<0.01).

Table S3. Heterogeneous impact results for regions at different levels of economic development

	**HFD**	**f_coi**	**n_coi**
	(1)	(2)	(3)
EcomP	2.423***<br>(0.950,3.896)	2.321***<br>(0.925,3.717)	0.882**<br>(0.090,1.674)
lnincome	-0.037<br>(-0.123,0.049)	-0.045<br>(-0.132,0.043)	0.050*<br>(-0.007,0.107)
lnincome×EcomP	-0.211**<br>(-0.379,-0.043)	-0.220***<br>(-0.378,-0.063)	-0.065<br>(-0.155,0.024)
Railper	-1.311<br>(-3.777,1.154)	-1.324<br>(-3.386,0.739)	-0.004<br>(-1.522,1.514)
female	0.045***<br>(0.018,0.072)	0.026*<br>(-0.002,0.055)	0.035***<br>(0.017,0.053)
School	-0.001***<br>(-0.002,-0.001)	-0.001***<br>(-0.002,-0.001)	-0.000*<br>(-0.001,0.000)
urban	0.003<br>(-0.003,0.009)	0.002<br>(-0.004,0.007)	0.003*<br>(-0.000,0.006)
Gini	0.000<br>(-0.004,0.004)	-0.001<br>(-0.005,0.003)	0.000<br>(-0.002,0.003)
foodcpi	-0.000<br>(-0.000,0.000)	0.000*<br>(-0.000,0.000)	0.000<br>(-0.000,0.000)
Hospital	-0.020**<br>(-0.039,-0.001)	-0.019*<br>(-0.041,0.003)	-0.010<br>(-0.024,0.003)
Industry	0.000<br>(-0.003,0.004)	0.000<br>(-0.003,0.003)	0.001<br>(-0.001,0.003)
Population	0.001<br>(-0.001,0.002)	0.001<br>(-0.000,0.001)	0.001***<br>(0.000,0.002)
agriopen	-0.161<br>(-0.622,0.299)	-0.178<br>(-0.631,0.274)	-0.078<br>(-0.406,0.251)
Agriculture	0.004<br>(-0.003,0.011)	0.001<br>(-0.006,0.008)	0.004*<br>(-0.001,0.009)
_cons	-1.183<br>(-3.286,0.920)	-0.141<br>(-2.234,1.953)	-1.762***<br>(-3.019,-0.504)
Country FE	Yes	Yes	Yes
Year FE	Yes	Yes	Yes
Observations	283	283	283
Adj R2	0.920	0.918	0.892

95% confidence interval (CI) are shown in parenthesis.
*denotes that the coefficient is significant at 10%(*p*<0.1); ** at 5%(*p*<0.05) and *** at 1%(*p*<0.01).
